# Higher Ratio of Serum Alpha-Fetoprotein Could Predict Outcomes in Patients with Hepatitis B Virus-Associated Hepatocellular Carcinoma and Normal Alanine Aminotransferase

**DOI:** 10.1371/journal.pone.0157299

**Published:** 2016-06-15

**Authors:** Young-Il Kim, Hyoung Sang Kim, Joong-Won Park

**Affiliations:** Center for Liver Cancer, National Cancer Center, Goyang, Korea; The University of Tokyo, JAPAN

## Abstract

**Background:**

The role of serum alpha-fetoprotein (AFP) levels in the surveillance and diagnosis of hepatocellular carcinoma (HCC) is controversial. The aim of this study was to investigate the value of serially measured serum AFP levels in HCC progression or recurrence after initial treatment.

**Methods:**

A total of 722 consecutive patients newly diagnosed with HCC and treated at the National Cancer Center, Korea, between January 2004 and December 2009 were enrolled. The AFP ratios between 4–8 weeks post-treatment and those at the time of HCC progression or recurrence were obtained. Multivariate logistic regression analysis was performed to correlate the post-treatment AFP ratios with the presence of HCC progression or recurrence.

**Results:**

The etiology of HCC was related to chronic hepatitis B virus (HBV) infection in 562 patients (77.8%), chronic hepatitis C virus (HCV) infection in 74 (10.2%), and non-viral cause in 86 (11.9%). There was a significant decrease in serum AFP levels from the baseline to 4 to 8 weeks after treatment (median AFP, 319.6 ng/mL vs. 49.6 ng/mL; p< 0.001). Multivariate analysis showed that an AFP ratio > 1.0 was an independently associated with HCC progression or recurrence. Among the different causes of HCC analyzed, this association was significant only for HCC related to chronic hepatitis B (p< 0.001) and non-viral causes (p<0.05), and limited only to patients who had normal alanine aminotransferase (ALT) levels.

**Conclusion:**

Serial measurements of serum AFP ratios could be helpful in detecting progression or recurrence in treated patients with HBV-HCC and normal ALT.

## Introduction

Serum alpha-fetoprotein (AFP) is recognized as a hepatocellular carcinoma (HCC)-specific marker [1], and older HCC guidelines included serum AFP measurement, combined with imaging studies, in the protocol for surveillance and diagnosis of HCC [2]. However, at the recommended cut-off value of 20 ng/mL, the sensitivity of serum AFP is significantly low [3]. A study analyzing prospectively collected data demonstrated that AFP was not an optimal test for the early detection of HCC among patients with hepatitis C [4]. Currently, AFP measurement is not included in Western guidelines for screening and diagnosis of HCC [5,6,7]. However, it is still included as a complementary tool for the screening and diagnosis of HCC in most Asian guidelines [7,8,9].

Despite a reduction in the importance of AFP as a screening and diagnostic test, there is evidence supporting its role as a prognostic marker in treated patients of HCC. High AFP level (1000 ng/mL) is associated with poor outcomes after liver transplantation [10], thus enabling effective selection of transplantation candidates [11] among patients with HCC. In addition, a recent study demonstrated that decrease in AFP levels in excess of 50% from the baseline value after locoregional treatments, including transarterial chemoembolization (TACE) or radioembolization by using yttrium-90, predicted better response to therapy, better survival, as well as early detection of progression [12]. The American Association for the Study of Liver Diseases (AASLD) guidelines identify an increase in post-treatment AFP levels as a sign of recurrence in patients with HCC, especially in those who had elevated pre-treatment serum AFP levels, and those who had normally decreased post-treatment AFP levels [6]. However, the efficacy of AFP monitoring is controversial and varies based on the etiology of chronic liver disease in patients with HCC [3,13]. In addition, the observed elevation of serum AFP may be owing to the underlying necrosis and inflammation [14].

Herein, we evaluated the role of higher post-treatment levels of AFP, as a marker of HCC progression or recurrence in treated patients of HCC, among the various etiologic groups.

## Patients and Methods

### Study population

**Fig 1:**
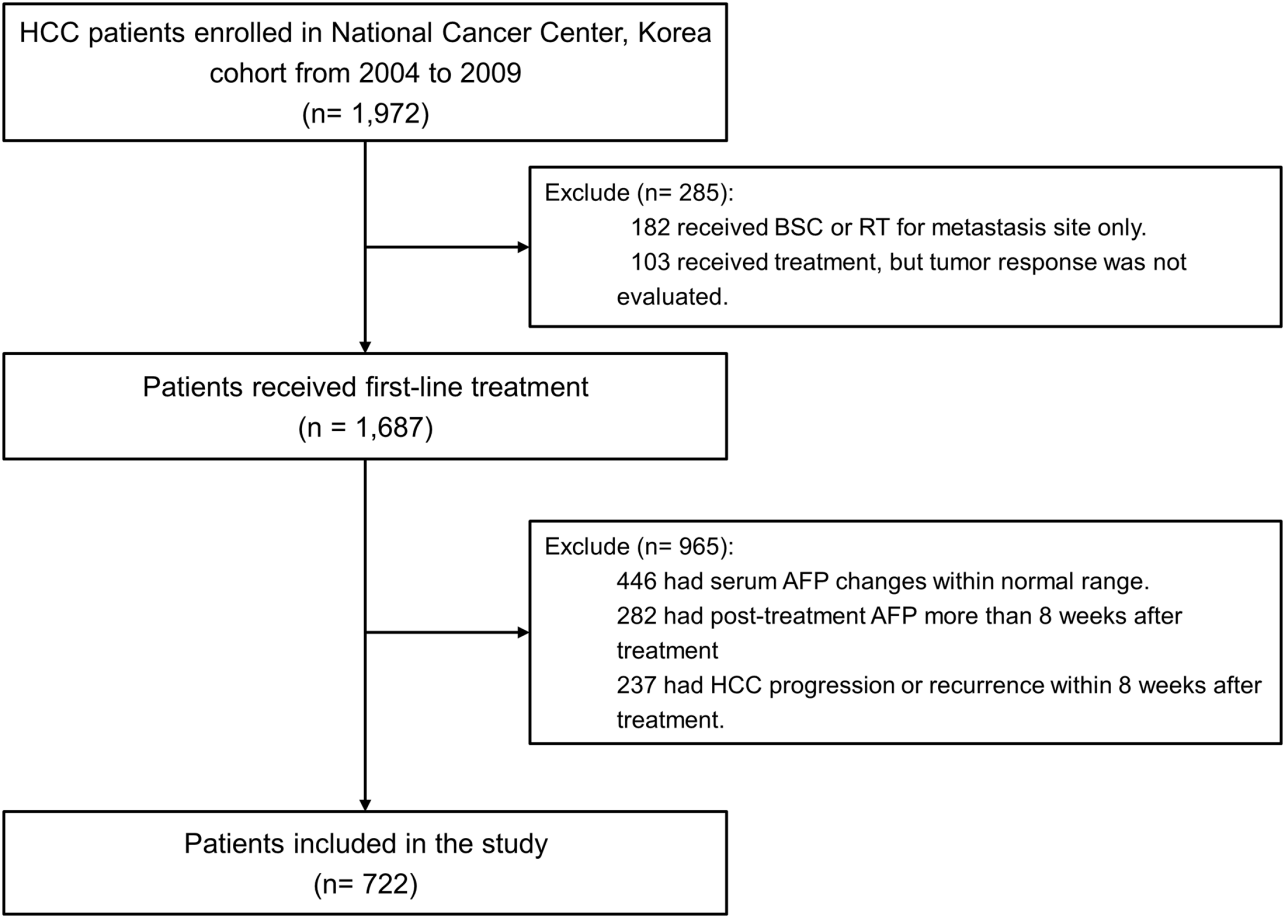
Study flows. Abbreviations: AFP, alpha-fetoprotein; BSC, best supportive care; HCC, hepatocellular carcinoma; RT, radiotherapy.

A total of 1,972 consecutive patients diagnosed with HCC were prospectively enrolled at the National Cancer Center, Korea from 2004 to 2009 and were considered for inclusion in the present study. The diagnosis of HCC was based on the guidelines of the Korean Liver Cancer Study Group and the National Cancer Center, Korea [7,15]. The guidelines were: 1) clinical and radiological criteria of early enhancement, followed by late wash-out on dynamic liver imaging such as computed tomography (CT) or magnetic resonance imaging (MRI) in conjunction with elevated levels of serum AFP, or 2) histological examination confirming HCC. Among the enrolled patients, 1,687 received first-line treatment and were evaluated for tumor response. Of which, 965 patients were excluded due to the following reasons: 446 patients had pre-treatment and post-treatment serum AFP levels within the normal range of 0–20 ng/mL, 282 patients had a delayed AFP measurement following treatment (more than 8 weeks), and 237 patients demonstrated early progression of HCC within 8 weeks of completing treatment. Thus, 722 patients were included in the final analyses (Fig 1).

All clinical data including baseline demographics and tumor characteristics were prospectively recorded. Initial tumor stage was evaluated by the Barcelona Clinic of Liver Cancer (BCLC) [16] as well as the modified International Union Against Cancer (mUICC) staging systems [17]. This study was approved by the Institutional Review Board of the National Cancer Center, Korea (IRB no. NCC2016-0017). The informed consents from all included patients were waived because of the lowest risk of the present study at the evaluation of the IRB.

### Tumor response and AFP analysis

Blood tests and dynamic CT or MRI images were verified to evaluate tumor response at 4–8 weeks following the initial treatment of HCC. The modified Response Evaluation Criteria in Solid Tumors (mRECIST) was used to evaluate tumor response. Response was accordingly categorized as complete response (CR), partial response (PR), stable disease (SD) and progressive disease (PD) [18]. Patients who achieved CR were followed up with imaging studies (dynamic CT or MRI) and blood tests, including AFP levels, every 3–4 months for the subsequent 2 years.

The AFP ratio was used to investigate whether serum AFP could diagnose HCC progression or recurrence following treatment. The AFP ratio was defined as follows: for patients who had HCC progression or recurrence, [the AFP ratio] = [AFP level at PD (progression or recurrence)] ÷ [AFP level at 4–8 weeks following the initial treatment], and for those who did not have HCC progression or recurrence, [the AFP ratio] = [AFP level at last follow-up] ÷ [AFP level at 4–8 weeks following the initial treatment].

### Statistical analyses

For categorical variables, data were presented as numbers and percentages, and for continuous variables, as median and interquartile range or mean and standard deviation. Chi-square or Fisher’s exact test was used for comparing categorical variables, and Mann-Whitney *U* test, one-way ANOVA or Kruskall-Wallis tests for continuous variables. Wilcoxon signed-rank test was used to compare the changes in AFP levels from baseline to post-treatment levels. To determine the optimal cut-off value of the AFP ratio in predicting HCC progression or recurrence, the area under receiver-operator characteristic (ROC) curves with 95% confidence intervals (CIs) was calculated. The optimal cut-off value was determined at an AFP level which had the highest the sum of sensitivity and specificity. Univariate and multivariate logistic regression analyses were performed to identify the risk factors associated with HCC progression after the initial treatment in patients who were categorized as CR, PR and SD based on treatment response. All data were analyzed by using STATA 13.1 (Stata Corp, College Station, TX, USA). A p-value of < 0.05 was considered statistically significant.

## Results

### Baseline clinical and tumor characteristics

The median age of the study population was 56 years (IQR, 49–64), and 574 patients (79.5%) were males. Patients were categorized based on the underlying etiology of HCC, as HBV group (562 patients, 77.8%), HCV group (74 patients, 10.3%) and non-viral group (86 patients, 11.9%). Patients in the HBV group were significantly younger than those in the HCV and non-viral groups. Patients in the non-viral group had significantly lower alanine aminotransferase (ALT) levels and larger tumor size than those in groups with viral etiology. Baseline AFP levels were similar in the HBV (median AFP, 372.5 ng/mL) and non-viral groups (median AFP, 640 ng/mL), and the two groups had significantly higher AFP levels than the HCV group (median AFP, 132.8 ng/mL) (p = 0.01). All groups were comparable with respect to the other baseline characteristics (Table 1).

**Table 1:** Baseline clinical and tumor characteristics according to the etiology.

	Etiology			p
	HBV	HCV	Non-viral	
	(n = 562)	(n = 74)	(n = 86)	
Age, in years (IQR)	53 (47–61)*^†^	65 (60–69)*	63 (54–70)^†^	0.001
Sex, n (%)				0.560
Female	115 (20.5)	18 (24.3)	15 (17.4)	
Male	447 (79.5)	56 (75.7)	71 (82.6)	
ALT (U/L), mean ± SD	63.2±113.3^†^	66.5±45.4^‡^	52.3±101.8^†^^‡^	<0.001
Baseline AFP (ng/mL)				
Median (IQR)	372.5 (57.2–3209.8)*	132.8 (35.6–1195.6)*^‡^	640.9 (70.1–5130.7)^‡^	0.010
Level category, n (%)				0.002
≤ 20.0	41 (7.30)	4 (5.4)	4 (4.7)	
20.1–200.0	190 (33.8)	43 (58.1)	29 (33.7)	
> 200.0	331 (58.9)	27 (36.5)	53 (61.6)	
Child class, n (%)				0.706
A	496 (88.3)	66 (89.2)	74 (86.1)	
B	61 (10.9)	7 (9.5)	12 (14.0)	
C	5 (0.9)	1 (1.4)	0 (0)	
MELD score, mean ± SD	7.9±3.7	8.2±4.0	7.2±4.3	0.117
Tumor size (cm), mean ± SD	5.3±3.8^†^	4.9±3.6^‡^	7.6±5.1^†^^‡^	0.001
BCLC stage, n (%)				0.096
A	167 (29.7)	19 (25.7)	18 (20.9)	
B	72 (12.8)	12 (16.2)	6 (7.0)	
C	323 (57.5)	43 (58.1)	62 (72.1)	
mUICC stage, n (%)				0.513
I	62 (11.0)	3 (4.1)	5 (5.8)	
II	197 (35.1)	27 (36.5)	28 (32.6)	
III	161 (28.7)	26 (35.1)	31 (36.1)	
IVa	96 (17.1)	12 (16.2)	16 (18.6)	
IVb	46 (8.2)	6 (8.1)	6 (7.0)	

Abbreviations: AFP, alpha-fetoprotein; BCLC, Barcelona Clinic of Liver Cancer; HBV, hepatitis B virus; HCV, hepatitis C virus; IQR, interquartile range; MELD, model for end-stage liver disease; mUICC, modified International Union Against Cancer; SD, standard deviation.

*p < 0.01 between HBV and HCV;

^†^p < 0.01 between HBV and non-viral;

^‡^p < 0.01 between HCV and non-viral

### Changes in serum AFP level 4–8 weeks following initial treatment of HCC

Median serum AFP levels were 319.6 ng/mL at baseline and 49.6 ng/mL, 4–8 weeks following the initial treatment of HCC, and this decrease in AFP levels was significant (p < 0.001). Post-treatment reduction in AFP levels were significant in patients who underwent resection (p < 0.001), liver transplantation (p = 0.002), radiofrequency ablation (RFA), percutaneous ethanol injection therapy (PEIT) (p < 0.001), or TACE (p < 0.001) as the initial treatment. However, patients receiving radiotherapy or chemotherapy (including sorafenib), had no significant changes in their post-treatment AFP levels. After the initial treatment, CR was documented in 343 patients (47.5%), PR in 114 patients (15.8%), SD in 227 patients (31.4%) and PD in 38 patients (5.3%). Significant decrease in AFP levels was demonstrated only in patients with CR, PR and SD (p < 0.001) (Table 2).

**Table 2:** Comparison of AFP levels at baseline and at 4–8 weeks following first-line treatment, based on treatment type and tumor response.

		AFP levels (ng/mL), median (IQR)		p*
	n, (%)	Baseline	4–8 weeks after treatments	
Total	722	319.6 (56.7–3022.1)	49.6 (14.6–429.2)	<0.001
Initial treatment				
Resection	153 (21.2)	532.0 (93.8–3482.9)	17.1 (7.4–58.1)	<0.001
Liver transplantation	13 (1.8)	82.3 (49.3–292.8)	3.1 (2.5–10.2)	0.002
RFA or PEIT	25 (3.5)	89.3 (34.5–477.6)	14.7 (9.3–22.3)	<0.001
TACE	489 (67.7)	234.4 (46.7–2761.5)	73.1 (20.6–576.6)	<0.001
RT	17 (2.4)	2504.7 (545.9–9896.4)	606.4 (346.8–10509.4)	0.227
Chemotherapy	17 (2.4)	19833.5 (5675.5–76295.8)	19919.5 (5295.1–71122.5)	0.831
Sorafenib	8 (1.1)	23593.6 (1678.2–168061.1)	32117.7 (2652.9–99847.0)	0.674
Tumor response				
Complete response	343 (47.5)	200.9 (44.2–1185.8)	19.5 (8.2–64.9)	<0.001
Partial response	114 (15.8)	241.5 (61.2–2432.4)	62.1 (19.3–673.3)	<0.001
Stable disease	227 (31.4)	769.0 (95.8–5555.4)	209.8 (47.6–2800.9)	<0.001
Progressive disease	38 (5.3)	7893.8 (1064.9–76295.8)	6771.5 (254.4–71122.5)	0.328

Abbreviations: AFP, alpha-fetoprotein; IQR, interquartile range; PEIT, percutaneous ethanol injection therapy; RFA, radiofrequency ablation; RT, radiotherapy; TACE, transarterial chemoembolization.

*Wilcoxon signed-rank test

### Performance of AFP ratio in prediction of HCC progression or recurrence

By the ROC curve analysis, AFP ratio predicted HCC progression or recurrence with statistical significance (area under the curve [AUC], 0.748; 95%CI, 0.70–0.79; p = 0.022; S1 Fig). In this analysis, the optimal cut-off value of AFP ratio in predicting HCC progression or recurrence was 1.1 (sensitivity 52.8%, specificity 87.0%, positive predictive value 94.8% and negative predictive value 29.0%).

### Factors associated with HCC progression after initial treatments

Older age, advanced mUICC stage, and higher AFP ratios (> 1.0) were associated with HCC progression on univariate analysis. On multivariate analysis, older age (adjusted odd ratio [aOR], 1.05; p < 0.001), mUICC stage III (aOR, 2.94; p = 0.005) and stage IV (aOR, 6.75; p < 0.001), and higher AFP ratio (aOR, 3.79 for AFP ratio of 1.1–2.0, p < 0.001; aOR, 17.47 for AFP ratio > 2.0, p < 0.001) were independent factors indicating HCC progression (Table 3).

**Table 3:** Factors associated with progression or recurrence in treated cases of hepatocellular carcinoma.

	No	Univariate analysis*			Multivariate analysis*		
		cOR	95% CI	p	aOR	95% CI	p
Age, years	684	1.04	1.02–1.06	<0.001	1.05	1.02–1.07	<0.001
Sex							
Male	541	1.00					
Female	143	0.67	0.43–1.03	0.07			
Etiology							
HBV	553	1.00					
HCV	72	1.72	0.83–3.56	0.147			
Non-viral	79	0.89	0.50–1.59	0.706			
Child class							
A	603	1.00					
B	75	1.42	0.72–2.77	0.310			
C	6	0.49	0.09–2.69	0.409			
Baseline AFP level							
≤ 20.0 ng/mL	48	1.00					
20.1–200.0 ng/mL	256	0.87	0.38–1.97	0.733			
> 200.0 ng/mL	380	0.81	0.37–1.81	0.613			
MELD score	684	1.05	0.99–1.11	0.052			
mUICC stage							
I	70	1.00			1.00		
II	251	0.70	0.39–1.27	0.243	0.65	0.33–1.25	0.197
III	210	3.12	1.55–6.28	0.001	2.94	1.38–6.25	0.005
IVa/ IVb	154	5.54	2.34–13.10	<0.001	6.75	2.71–16.83	<0.001
Ratio of AFP level							
≤ 1.0	377	1.00			1.00		
1.1–2.0	119	4.26	2.20–8.22	<0.001	3.79	1.89–7.59	<0.001
> 2.0	188	13.15	5.66–30.53	<0.001	17.47	7.34–41.54	<0.001

Abbreviations: AFP, alpha-fetoprotein; aOR, adjusted odd ratio; CI, confidence interval; cOR, crude odd ratio; HBV, hepatitis B virus; HCV, hepatitis C virus; MELD, model for end-stage liver disease; mUICC, modified International Union Against Cancer; PD, progressed disease.

*Logistic regression model was used for univariate and multivariate analyses.

#### The AFP ratio at the time of HCC progression or recurrence according to ALT levels

During a median follow-up period of 8.8 months (IQR, 4.6–21.6) between the initial treatment and HCC progression, 553 patients (80.8%), among the 684 who were categorized as CR, PR or SD following treatment, were found to have HCC progression. AFP ratio was significantly higher in patients who had progression (median ratio, 1.1) than in those without progression (median ratio, 0.4) (p < 0.001). In addition, patients with progression were more likely to have an AFP ratio of greater than 1.0 (p < 0.001) than the others. The difference in the AFP ratio among patients with progressive and non-progressive HCC was significant in the HBV and non-viral groups, but not in the HCV group. In addition, this association between HCC progression and AFP ratio was significant only in patients with ALT levels ≤ 40 U/L (Table 4).

**Table 4:** Correlation of AFP ratios with HCC progression based on serum ALT levels.

	Etiology											
	Total (n = 684)			HBV (n = 533)			HCV (n = 72)			Non-viral (n = 79)		
	Progression			Progression			Progression			Progression		
	No	Yes	p	No	Yes	p	No	Yes	p	No	Yes	p
Total (n = 684)	n = 131	n = 553		n = 105	n = 428		n = 9	n = 63		n = 17	n = 62	
AFP ratio, median (IQR)	0.4 (0.1–0.9)	1.1 (0.5–3.0)	<0.001*	0.4 (0.1–0.9)	1.1 (0.5–3.1)	<0.001*	0.6 (0.2–1.2)	1.3 (0.5–2.7)	0.192*	0.3 (0.2–0.6)	1.1 (0.3–2.7)	0.001*
AFP ratio, n (%)			<0.001^†^			<0.001^†^			0.593^†^			0.002^†^
≤ 1.0	114 (30.2)	263 (69.8)		92 (31.1)	204 (68.9)		6 (17.7)	28 (82.4)		16 (34.0)	31 (66.0)	
1.1–2.0	11 (9.2)	108 (90.8)		8 (9.4)	77 (90.6)		2 (10.0)	18 (90.0)		1 (7.1)	13 (92.9)	
> 2.0	6 (3.1)	182 (96.8)		5 (3.3)	147 (96.7)		1 (5.6)	17 (94.4)		0 (0)	18 (100)	
ALT ≤ 40 U/L (n = 458)	n = 108	n = 350		n = 89	n = 262		n = 4	n = 37		n = 15	n = 51	
AFP ratio, median (IQR)	0.3 (0.1–0.8)	1.1 (0.5–3.0)	<0.001*	0.3 (0.1–0.7)	1.1 (0.5–3.3)	<0.001*	0.2 (0.1–0.5)	1.0 (0.5–2.6)	0.077*	0.3 (0.1–0.8)	1.0 (0.2–1.7)	0.006*
AFP ratio, n (%)			<0.001^†^			<0.001^†^			0.373^†^			0.021^†^
≤ 1.0	101 (37.0)	172 (63.0)		83 (39.9)	125 (60.1)		4 (17.4)	19 (82.6)		14 (33.3)	28 (66.7)	
1.1–2.0	6 (8.6)	64 (91.4)		5 (10.0)	45 (90.0)		0 (0)	8 (100)		1 (8.3)	11 (91.7)	
> 2.0	1 (0.9)	114 (99.1)		1 (1.1)	92 (98.9)		0 (0)	10 (100)		0 (0)	12 (100)	
ALT > 40 U/L (n = 226)	n = 23	n = 203		n = 16	n = 166		n = 5	n = 26		n = 2	n = 11	
AFP ratio, median (IQR)	0.9 (0.3–2.0)	1.2 (0.5–3.0)	0.223*	1.0 (0.3–3.8)	1.1 (0.5–2.9)	0.471*	1.2 (0.6–2.0)	1.5 (0.7–2.7)	0.753*	0.3 (0.2–0.4)	2.7 (1.0–4.5)	0.086*
AFP ratio, n (%)			0.470^†^			0.838^†^			0.944^†^			0.269^†^
≤ 1.0	13 (12.0)	91 (87.5)		9 (10.2)	79 (89.8)		2 (18.2)	9 (81.8)		2 (40.0)	3 (60.0)	
1.1–2.0	5 (10.2)	44 (89.8)		3 (8.6)	32 (91.4)		2 (16.7)	10 (83.3)		0 (0)	2 (100)	
> 2.0	5 (6.9)	68 (93.2)		4 (6.8)	55 (93.2)		1 (12.5)	7 (78.5)		0 (0)	6 (100)	

Abbreviations: AFP, alpha-fetoprotein; HBV, hepatitis B virus; HCV, hepatitis C virus; IQR, interquartile range.

*Mann-Whitney U test;

^†^Fisher’s exact test

Subgroup analysis was performed only in the 343 patients who demonstrated CR after the initial treatment to evaluate the association between AFP ratio and HCC recurrence. In these patients, the AFP ratio was significantly higher in patients with HCC recurrence than in those without recurrence (p < 0.001). When analyzed separately based on HCC etiology, this significance persisted only for the HBV group (p <0.001). The non-viral group also demonstrated a higher AFP ratio at the time of HCC recurrence, though this was not statistically significant (p = 0.052). The proportion of patients with AFP ratio more than 1.0 was significantly higher in patients with HCC recurrence (p < 0.001); HBV and non-viral groups had a significantly higher proportion of patients with AFP ratio greater than 1.0 (HBV group, p <0.001; non-viral group, p = 0.007). There was no such correlation in the HCV group (p = 0.859). In the subgroup analyses, the association between higher AFP ratio and HCC recurrence was significant only in patients with normal ALT levels at recurrence (Table 5).

**Table 5:** Correlation of AFP ratios with HCC progression based on serum ALT levels in patients who achieved complete response (CR).

	Etiology											
	Total (n = 343)			HBV (n = 271)			HCV (n = 33)			Non-viral (n = 39)		
	Recurrence			Recurrence			Recurrence			Recurrence		
	No	Yes	p	No	Yes	p	No	Yes	p	No	Yes	p
Total (n = 343)	n = 108	n = 235		n = 85	n = 186		n = 8	n = 25		n = 15	n = 24	
AFP ratio, median (IQR)	0.3 (0.1–0.7)	1.2 (0.5–5.7)	<0.001*	0.3 (0.1–0.7)	1.3 (0.5–7.0)	<0.001*	0.5 (0.2–1.4)	1.0 (0.6–1.8)	0.146*	0.2 (0.1–0.6)	1.0 (0.1–4.3)	0.052*
AFP ratio, n (%)			<0.001^†^			<0.001^†^			0.859^†^			0.007^†^
≤ 1.0	98 (47.3)	109 (52.7)		78 (49.1)	81 (50.9)		6 (30.0)	14 (70.0)		14 (50.0)	14 (50.0)	
1.1–2.0	7 (19.4)	29 (80.6)		5 (17.9)	23 (82.1)		1 (16.7)	5 (83.3)		1 (50.0)	1 (50.0)	
> 2.0	3 (3.0)	97 (97.0)		2 (2.4)	82 (97.6)		1 (14.3)	6 (85.7)		0 (0)	9 (100)	
ALT ≤ 40 U/L (n = 256)	n = 95	n = 161		n = 77	n = 125		n = 4	n = 16		n = 14	n = 20	
AFP ratio, median (IQR)	0.2 (0.1–0.6)	1.2 (0.5–5.7)	<0.001*	0.2 (0.1–0.6)	1.4 (0.5–7.4)	<0.001*	0.2 (0.1–0.5)	1.0 (0.4–2.9)	0.025*	0.3 (0.1–0.6)	1.0 (0.2–4.3)	0.053*
AFP ratio, n (%)			<0.001^†^			<0.001^†^			0.624^†^			0.033^†^
≤ 1.0	90 (54.2)	76 (45.8)		73 (57.5)	54 (42.5)		4 (28.6)	10 (71.4)		13 (52.0)	12 (48.0)	
1.1–2.0	5 (22.7)	17 (77.3)		4 (21.1)	15 (79.0)		0 (0)	1 (100)		1 (50.0)	1 (50.0)	
> 2.0	0 (0)	68 (100)		0 (0)	56 (100)		0 (0)	5 (100)		0 (0)	7 (100)	
ALT > 40 U/L (n = 87)	n = 13	n = 74		n = 8	n = 61		n = 4	n = 9		n = 1	n = 4	
AFP ratio, median (IQR)	0.6 (0.3–2.0)	1.2 (0.5–5.3)	<0.001*	0.7 (0.3–9.8)	1.2 (0.5–7.0)	0.399*	1.3 (0.5–2.9)	1.1 (0.7–1.4)	0.938*	0.2 (0.2–0.2)	1.4 (0.1–4.5)	>0.999*
AFP ratio, n (%)			0.511^†^			0.676^†^			>0.999^†^			>0.999^†^
≤ 1.0	8 (19.5)	33 (80.5)		5 (15.6)	27 (84.4)		2 (33.3)	4 (66.7)		1 (33.3)	2 (66.7)	
1.1–2.0	2 (14.3)	12 (85.7)		1 (11.1)	8 (88.9)		1 (20.0)	4 (80.0)		-	-	
> 2.0	3 (9.4)	29 (90.6)		2 (7.1)	26 (92.9)		1 (50.0)	1 (50.0)		0 (0)	2 (100)	

Abbreviations: AFP, alpha-fetoprotein; HBV, hepatitis B virus; HCV, hepatitis C virus; IQR, interquartile range.

*Mann-Whitney U test;

^†^Fisher’s exact test

## Discussion

Serum AFP levels are no longer universally accepted for inclusion in protocols for surveillance and diagnosis of HCC. Although one guideline recommended a combination of serial AFP measurements and imaging studies to monitor the progression of HCC in treated patients [6], evidence of its efficacy was not well studied. In our study, the association of higher AFP ratios (> 1.0) with disease progression or recurrence was significant only in cases of HCC secondary to hepatitis B or non-viral causes, and this association was limited to patients who had normal ALT levels. In addition, higher AFP ratios were independent factors associated with HCC progression after initial treatments in large number of HCC patients. Our findings suggest that serial measurement of serum AFP levels may be helpful in detecting recurrence or progression of disease in patients with HBV-associated HCC provided patients have normal ALT levels. This may be achieved due to immune tolerance stage or nucleotide/side analogue treatment.

Two studies reported the role of post-treatment AFP levels in predicting tumor recurrence and outcomes [12,19]. One study included a small number of patients who underwent only loco-regional therapy [12]. The other study analyzed 57 patients and showed correlation between AFP levels and the size of the recurrent tumor [19]. Our study included a large cohort of patients with prospectively collected data that were retrospectively analyzed. This study design has a reduced selection bias than a retrospective study. However, it should be noted that 282 patients (282/1687, 16.7%) in the cohort missed the scheduled follow-up test at 4–8 weeks after initial treatment.

Several previous studies also demonstrated the prognostic roles of baseline [20–22] and post-treatment AFP levels [12,19]. High serum AFP levels at the time of diagnosis of HCC were associated with worse clinicopathologic features and poor outcomes; larger tumor size, bilobar involvement, massive or diffuse tumor types, portal vein thrombosis, advanced stages, early recurrence and poor overall survival [20–22]. In addition, post-treatment reductions in AFP levels were associated with better clinical outcomes [12,19]. In the present study, we also found that increased AFP ratio from 4 to 8 weeks after initial treatments was an independent risk factor for HCC progression. Thus, serial AFP measurements in treated HCC patients may be a useful method of monitoring for recurrence.

In patients with hepatitis B or C virus infection, measurements of serum AFP levels were suboptimal in detecting HCC [3] as the AFP levels were frequently elevated even in the absence of HCC in patients with chronic hepatitis B [23] or C [24]. However, serum AFP levels decreased following anti-viral therapy in patients with chronic hepatitis B, and in those patients who demonstrated elevated AFP levels after anti-viral therapy, the risk of HCC was high [25]. In the present study, AFP ratio > 1.0 was significantly associated with HCC progression only in the HBV and non-viral groups, but not in the HCV group. These findings suggest that post-treatment serum AFP measurements may be helpful in detecting progression or recurrence in HCC secondary to chronic hepatitis B or non-viral causes. Since this is the era of direct-acting drug therapy for chronic hepatitis C, further studies are warranted to define the role of AFP in patients with HCV-associated HCC and direct-acting drug treatment.

Serum ALT levels, which reflect chronic liver disease activity, have an association with serum AFP levels [3,14,26]. In the present study, the positive association between high AFP ratio and HCC progression was valid only in patients with normal post-treatment ALT levels. These results suggest that the value of AFP as a marker of progressive or recurrent HCC is limited to patients with normal serum ALT levels.

The main strength of the present study is the large cohort (n = 722) of patients analyzed. However, this study had several limitations. First, the present study was a retrospective analysis, which may have introduced selection bias. Second, the HCV and non-viral groups had far fewer patients than the HBV group. This could not be avoided owing to the preponderance of HBV infection as the main cause (around 75%) for HCC in South Korea.

In conclusion, AFP ratios greater than 1.0 in treated cases of HCC were significantly associated with HCC progression or recurrence. However, among the different causes of HCC analyzed, this association was significant only for HCC related to chronic hepatitis B and non-viral causes, and limited only to patients who had normal ALT levels. AFP ratios did not aid in the detection of recurrence in HCV associated HCC. Our results suggest that serial measurement of serum AFP in treated patients of HBV-associated HCC may be a useful strategy for the detection of disease progression or recurrence. Further controlled prospective studies are required to confirm this finding.

## Supporting Information

S1 Fig ROC curve for alpha-fetoprotein ratio in prediction of hepatocellular carcinoma progression or recurrence. (TIF)

## Abbreviations

AFP: alpha-fetoprotein
HCC: hepatocellular carcinoma
ALT: alanine aminotransferase
HBV: hepatitis B virus
HCV: hepatitis C virus
