# Supplementary figures and images for: Higher Ratio of Serum Alpha-Fetoprotein Could Predict Outcomes in Patients with Hepatitis B Virus-Associated Hepatocellular Carcinoma and Normal Alanine Aminotransferase
Source: PLoS One. 2016 Jun 15;11(6):e0157299. doi: 10.1371/journal.pone.0157299 (PMC4909194; doi:10.1371/journal.pone.0157299)

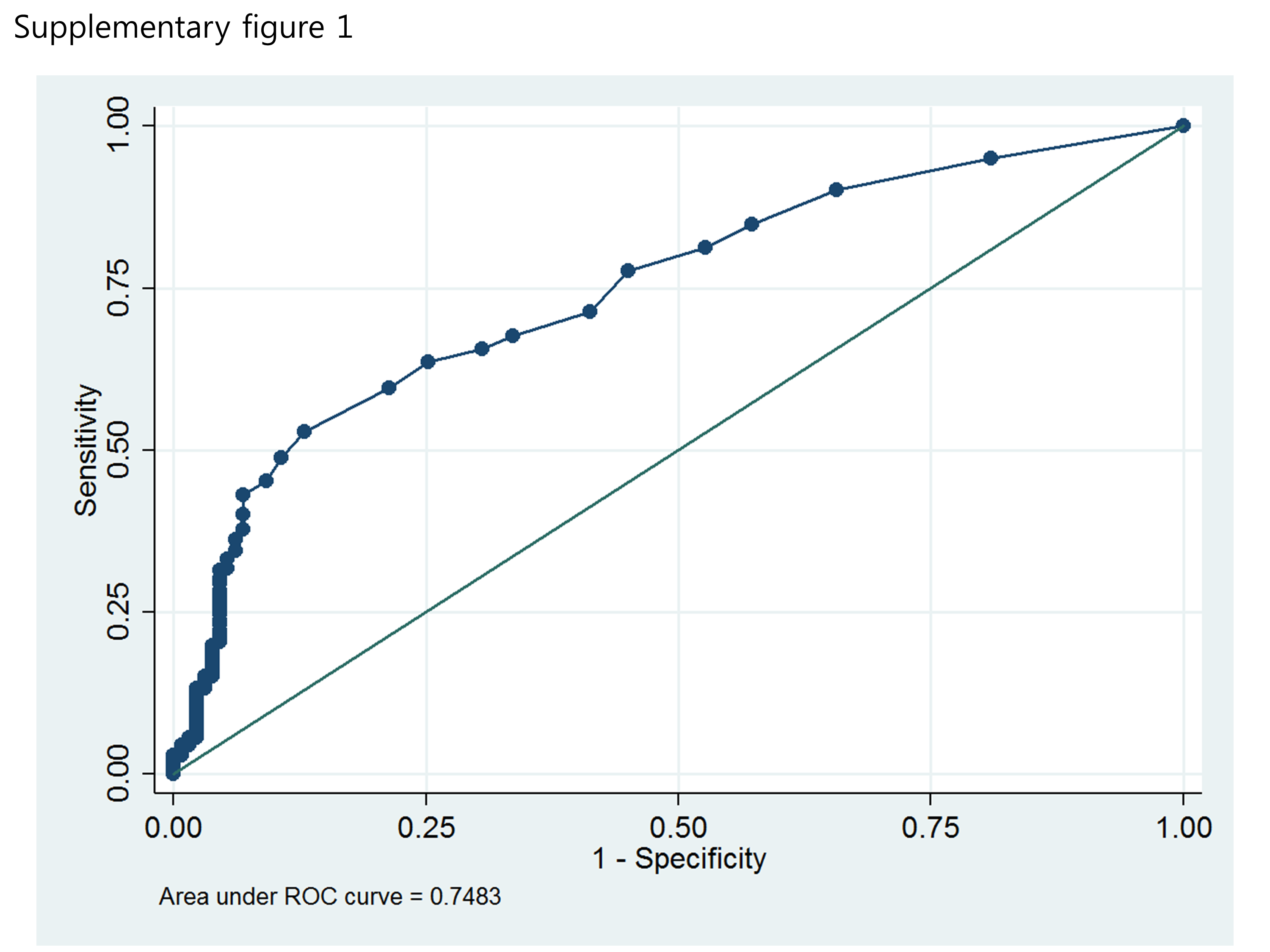

Supplement: S1 Fig — (TIF) [file pone.0157299.s001.tif]
